# Mesenchymal Stem Cells (MSCs): A Novel Therapy for Type 2 Diabetes

**DOI:** 10.1155/2022/8637493

**Published:** 2022-08-22

**Authors:** Shuang Gao, Yuanyuan Zhang, Kaini Liang, Ran Bi, Yanan Du

**Affiliations:** Department of Biomedical Engineering, School of Medicine, Tsinghua-Peking Center for Life Sciences, Tsinghua University, Beijing 100084, China

## Abstract

Although plenty of drugs are currently available for type 2 diabetes mellitus (T2DM), a subset of patients still failed to restore normoglycemia. Recent studies proved that symptoms of T2DM patients who are unresponsive to conventional medications could be relieved with mesenchymal stem/stromal cell (MSC) therapy. However, the lack of systematic summary and analysis for animal and clinical studies of T2DM has limited the establishment of standard guidelines in anti-T2DM MSC therapy. Besides, the therapeutic mechanisms of MSCs to combat T2DM have not been thoroughly understood. In this review, we present an overview of the current status of MSC therapy in treating T2DM for both animal studies and clinical studies. Potential mechanisms of MSC-based intervention on multiple pathological processes of T2DM, such as *β*-cell exhaustion, hepatic dysfunction, insulin resistance, and systemic inflammation, are also delineated. Moreover, we highlight the importance of understanding the pharmacokinetics (PK) of transplanted cells and discuss the hurdles in MSC-based T2DM therapy toward future clinical applications.

## 1. Introduction

Diabetes mellitus (DM) consists of an array of dysfunctions characterized by hyperglycemia and has become one of the most prevalent chronic diseases worldwide. Diabetes has afflicted more than 436 million people in 2019, and this number is estimated to reach 700 million by 2045. Type 1 diabetes mellitus (T1DM) is caused by a deficiency of insulin production, while type 2 diabetes mellitus (T2DM) is linked to insulin resistance. Precisely, more than 90% of diabetic patients are affected by T2DM and, to a large extent, associated with obesity, lack of exercise, poor diet, and heredity [1, 2]. Insulin resistance occurs when cells in the muscle, adipose tissue, and liver insensitively respond to the action of insulin, thus engendering numerous pathogeneses that encompass the accumulation of ectopic lipid metabolites, activation of unfolded protein response (UPR) pathways, and activation of innate immune pathways [2]. Insulin resistance is primarily compensated by elevated insulin secretion, which eventually leads to T2DM due to the exhaustion of pancreatic *β*-cells [3]. Therefore, insulin resistance has become the most prominent predictor of T2DM progression, as well as a potential therapeutic target once hyperglycemia is present [4].

Besides hyperglycemia, most diabetic patients are apt to suffer from various life-threatening complications (e.g., cardiovascular diseases and stroke) that reduce their quality of life and could even inflict fatal outcomes, which further highlights the necessity of suitable pharmacological intervention for the prevention and treatment of diabetes. In conformity with the American Diabetes Association (ADA), the regular treatment of T2DM is based on lifestyle interventions, including a healthy diet, weight loss, and regular practice of physical activity [5]. Nonetheless, these efforts should be performed in concert with antidiabetic drugs for consolidated maintenance of normoglycemia. To date, eight classes of antidiabetic drugs have been approved by the Food and Drug Administration (FDA), including the first-line drug metformin and newly developed glucagon-like peptide-1 receptor agonists (GLP-1RAs) [6], along with versatile medication protocols such as monotherapy, dual therapy, and multiagent therapy to improve the efficacy of T2DM treatment [7]. However, certain pathologies of T2DM, such as *β*-cell exhaustion, hepatic dysfunction, insulin resistance, and systemic inflammation, remain refractory with the employment of conventional medications. Besides, these medications are associated with a myriad of risks and side effects, including hypoglycemia, diarrhea, and liver damage, signifying the indispensability of developing an antidiabetic drug ideal for the augmentation of insulin sensitivity and reversal of pancreatic *β*-cell failure [7].

Recently, cell-based therapies have emerged as the next-generation medicine to address intricate physiopathologies of T2DM [8–10]. Mesenchymal stem/stromal cells (MSCs) have demonstrated their therapeutic effects in both animal studies and clinical studies, thus offering adept modalities in treating T2DM. In brief, MSCs are capable of self-renewal and differentiating into multiple mesenchymal lineages, such as adipogenic, chondrogenic, and osteogenic lineages *in vitro*. Moreover, they exhibit low immunogenicity due to the intermediate expression of major histocompatibility complex (MHC) class I, as well as the absence of MHC class II and costimulatory molecules on their cell surfaces [11, 12]. Besides, the plethora of cytokines, growth factors, and exosomes secreted by MSCs play a pivotal role in the regulation of insulin sensitivity and *β*-cell dysfunction [13, 14]. Most significantly, previous studies have indicated that MSCs are capable of exerting certain antidiabetic effects, as supported by the evidence that multiple infusions of MSCs may reverse hyperglycemia instead of single-dose infusion [15, 16]. In this review, we summarize various animal and clinical studies of MSC therapy in treating T2DM. Next, we shed light on the possibility of MSC-based therapy as a novel antidiabetic treatment, with a focus on its potential therapeutic mechanisms. Finally, critical challenges toward the clinical translation of MSC therapy for T2DM are discussed through the viewpoint of cellular pharmacokinetics (PK) and safety considerations.

## 2. Preclinical Studies of MSCs for T2DM Treatment

The multiplexed ability of MSCs to ameliorate T2DM-associated metabolic syndromes such as hyperglycemia, insulin resistance, and systemic inflammation has heretofore been delineated by numerous animal studies. The MSC sources, animal models, delivery routes, and interventions used in these research studies have been summarized in Table 1. Briefly, the main sources of MSCs include the umbilical cord, adipose tissue, and bone marrow from autologous, allogeneic, and xenogeneic origins [17–19]. Interestingly, several publications that involved human-derived MSCs revealed that xenogeneic cells conferred suboptimal therapeutic effects in T2DM animal models and did not lead to severe graft rejection [17, 19–21].

Furthermore, the most widely used T2DM models in these studies can be stratified into the high-fat diet- (HFD-) induced model, fat-fed/streptozocin- (STZ-) induced model, and leptin receptor-deficient (db/db) model. However, the duration of obesity and T2DM induction varies between different studies, thus causing different pathological stages of T2DM. Generally, a longer time is needed to induce *β*-cell dysfunction than insulin resistance and hyperlipidemia, while 30 weeks were taken to induce nonalcoholic steatohepatitis (NASH) syndrome in small animals [18]. Moreover, STZ is usually injected intrapancreatically into animals after 10 weeks of HFD treatment in order to accelerate the induction of *β*-cell dysfunction [19, 22]. Besides, the db/db model, which is characterized by the deficiency in leptin receptors, is also well adopted owing to the steadily high plasma glucose level [23].

According to Table 1, the majority of researchers deliver therapeutic MSCs through the intravenous tail vein despite the fact that MSCs would be trapped within the lung capillaries and eliminated rapidly within hours postadministration [18, 22–25]. On the other hand, a single administration of MSCs has been proved to provide potent therapeutic effects on glucose tolerance and insulin tolerance in diabetic animals [18, 22]. However, only a limited number of articles summarize the versatile therapeutic effects of MSCs among diverse formulation and dosing regimens on T2DM animal models. Therefore, further studies should be carried out to establish the standard guidelines to be implemented in MSC therapy.

In addition to optimizing the MSC sources, animal models, administration routes, and dosages, cell engineering strategies have been scrutinized to improve the therapeutic outcomes of MSCs. In particular, genetically modified MSCs were exploited to induce the secretion of rarely expressed or nonnative therapeutic proteins with the advent of gene-editing tools such as CRISPR-Cas9, viral and nonviral vectors. For instance, Xu et al. have exemplified that the overexpression of insulin-producing genes in mouse MSCs significantly sustained their antidiabetic effects *in vivo* after intrahepatic administration [26]. Karnieli et al. also reported that MSCs transfected with pancreatic and duodenal homeobox-1 (PDX-1) can reduce blood glucose in STZ-diabetic severe combined immunodeficient (SCID) mice after 5 weeks [27], accompanied with some drawback as the mice developed abnormal glucose tolerance after 6-8 weeks of transplantation. In addition, Milanesi et al. used human bone marrow mesenchymal stem cells (hBM-MSCs) to coexpress the vascular endothelial growth factor (VEGF) and PDX-1 transiently and were able to reverse hyperglycemia in more than half of the diabetic mice, denoting that MSCs improved their overall survival and body weight [28]. However, discrepant effects were observed between mice treated with hBM-MSCs with dual and single gene expressions. Aside from insulin-producing genes, PDX-1, VEGF, and interfering neurogenin 3 (Ngn3) have also been integrated into MSCs to augment their antidiabetic effects [29]. In our recent research, we genetically engineered MSCs with Exendin-4 (MSC-Ex-4) and demonstrated their boosted cellular function and antidiabetic efficacy in the T2DM mouse model. The Exendin-4 secreted by MSC-Ex-4 improved MSC survival under high glucose stress via autocrine activation of the GLP-1R-mediated AMPK signaling pathway, as well as suppressed senescence and apoptosis of pancreatic *β*-cells through endocrine effects. We also showed that the amplified secretion of bioactive factors (e.g., IGFBP2 and APOM) of MSC-Ex-4 paracrinely augments insulin sensitivity and decreases lipid accumulation in hepatocytes through PI3K-AKT activation [30]. Indisputably, the functional proteins secreted by genetically modified MSCs may be useful to mitigate NASH and metabolic-associated fatty liver disease (MAFLD) concomitantly, concerning that diabetes is intimately associated with these complications. In concise detail, the antidiabetic GLP-1RA commercialized by Novo Nordisk, namely, semaglutide, has shown encouraging effects in resolving the symptoms of NASH in phase 2 trials [31].

Nevertheless, these genetically engineered MSCs still exhibited numerous setbacks, which lead to the underwhelming therapeutic effects of MSCs. Firstly, transient transfection is extremely unstable, thus resulting in short-lasting therapeutic effects. Secondly, most viral vectors are not desirable in clinical settings due to the possibility of causing carcinogenesis and immune responses, which indicates a demand for other cell engineering modalities in order to enhance the MSC potency. Simultaneously, maintaining the low generation of MSCs and reducing the cell damage during cell transfection and chemical (e.g., puromycin) selection are challenging tasks. Meanwhile, the ethical concerns involving gene manipulation face a considerable degree of skepticism. However, with the continuous advancement of gene-editing tools with unprecedented spatiotemporal control, we believe that the genetic manipulation techniques are prompt to have enhanced precision, efficacy, and safety [32, 33].

## 3. Clinical Studies of MSCs for T2DM Treatment

According to the data published by the National Institutes of Health (NIH), current clinical trials of MSCs involved in the treatment of diabetes mainly focus on T1DM patients. In 2008, the University of Miami has started MSC therapy on T2DM patients by using bone marrow stem cells (BM-SCs), which were harvested from the patient's iliac crest bone marrow [38]. Although this study did not authenticate the identity of isolated cells, most of these cells were claimed to be MSCs according to the isolation method. The metabolic panels showed significant improvement in T2DM patients when comparing baseline data with 12 months of follow-up data. Furthermore, combinatorial therapy of intrapancreatically infused autologous stem cells (ASCs) and hyperbaric oxygen therapy (HBO) can improve the metabolic and insulin control of T2DM patients. Still, further randomized and controlled clinical trials are necessary to validate these findings.

According to Table 2, although human umbilical cord mesenchymal stem cells (hUC-MSCs), BM-MSCs, and bone marrow mononuclear cells (BM-MNCs) are the mostly used cell types in clinical trials, some infrequently used cell types or conditions, such as hypoxia preconditioned mesenchymal stem cells (HP-MSCs) and bone marrow-derived mesenchymal precursor cells (BM-MPCs), also show their therapeutic effects. Besides, MSC therapy was applied as an adjuvant to strengthen the efficacy of antidiabetic drugs. In a Chinese clinical trial, 12 T2DM patients who failed to reinstate normal glycemic control after liraglutide treatment were treated with 1 × 10^6^ cells/kg of hUC-MSCs via pancreatic artery infusion on the first day, with another 1 × 10^6^ cells/kg of cells infused through the peripheral vein on days 8, 15, and 22. On the contrary, control subjects were infused with saline, while both groups were treated with liraglutide for 24 weeks. The result demonstrated that the fasting plasma glucose (FPG), postload glucose (2hPG), and hemoglobin A1c (HbA1c) levels were significantly decreased in subjects who received MSC therapy in comparison with control groups [39], indicating that MSCs can improve glucose metabolism and *β*-cell function in T2DM patients in combination with other medications or therapies.

In addition, intrapancreatic and intravenous infusion methods are generally used in clinical studies due to safety concerns. In 2014, Liu et al. found that subcutaneous hematoma was developed at the injection site of a patient during the first day of intrapancreatic injection, which was resolved subsequently after seven days. Besides, nausea, vomiting, and headache also occurred in another patient, who recovered spontaneously within one week. Therefore, although previous clinical trials showed that intravenously injected MSCs can cause pulmonary microembolism, no serious adverse reactions have been indicated so far [40]. In addition, the therapeutic effects of MSCs can be enhanced when combined with biological materials, such as collagen and hydrogels. A clinical study that was aimed at improving the erectile function of men with diabetes by the injection of collagen hydrogel and hUC-MSC mixture into the cavernous body was recruiting in 2015. Since a collagen scaffold has been demonstrated to prolong the lifetime and maintain the stemness of MSCs, we can assume that the combination of stem cell therapy and tissue engineering can further augment the therapeutic efficacy of MSCs.

## 4. The Mechanisms of MSC Therapy in T2DM

Although the therapeutic efficacy of MSC therapies for T2DM has been postulated decades ago, their underlying mechanisms remain elusive. Therefore, multiple potential mechanisms of MSCs in various pathological processes of T2DM, such as *β*-cell exhaustion, hepatic dysfunction, insulin resistance, and systemic inflammation, are envisaged here.

### 4.1. *β*-Cell Regeneration

MSCs promote insulin production by facilitating the regeneration of endogenous pancreatic islet *β*-cells, and several hypotheses about their fundamental mechanisms have been reported. Although previous studies have proved that MSCs can differentiate into *β*-cells or insulin-producing cells *in vitro* [48–50], it is increasingly evidenced that limited transdifferentiation of the infused MSCs could occur *in vivo* to facilitate the process of pancreas regeneration and ameliorate hyperglycemia in T2DM models. For example, Hess et al. discovered that despite the elevated insulin production of streptozotocin- (STZ-) induced mice at 42 days after the intravenous injection of hBM-MSCs, the majority of the transplanted cells migrated to ductal and islet structures, and only a minority of transplanted cells are labeled with insulin [51]. Therefore, although MSCs can initiate endogenous insulin production and stimulate the proliferation of *β*-cells, transdifferentiation of MSCs into *β*-cells and transplantation engraftment may not significantly contribute to the restoration of pancreas function.

**Figure 1:**
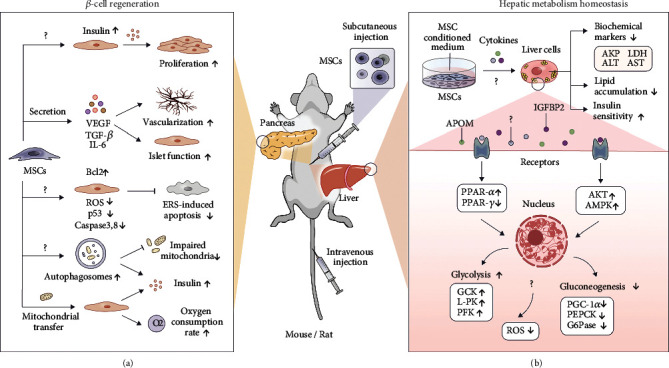
(a) Possible mechanisms of action for MSCs to promote islet regeneration. MSCs might initiate endogenous insulin production and stimulate the proliferation of *β*-cells. Various bioactive molecules secreted by MSCs, such as VEGF, TGF-*β*, and IL-6, can lead to enhanced vascularization and islet function. Besides, mitochondria of MSCs could be transferred to *β*-cells under hypoxia conditions to enhance the insulin secretion rate. MSCs show their antiapoptotic effect by downregulating ROS, caspase 3, caspase 8, and p53 and upregulating Bcl2. MSCs are capable of enhancing the formation of phagosomes, leading to the improved clearance of impaired mitochondria and the increased number of insulin granules. (b) Possible mechanisms of action for MSCs to influence hepatic metabolic homeostasis. MSCs can reduce the number of impaired mitochondria and systemic ROS levels to prevent liver metabolic imbalance. Upon MSC administration, PPAR-*α* was upregulated while PPAR-*γ* was downregulated. The expression of enzymes involved in hepatic glycolysis (GCK, L-PK, and PFK) is elevated, while the enzymes involved in gluconeogenesis (PGC-1*α*, PEPCK, and G6Pase) are reduced. In addition, MSCs can activate AKT and AMPK signaling pathways, which play a key role in cell metabolism. MSCs could significantly lower disordered biochemical markers of liver function caused by HFD, for instance, AKP, LDH, ALT, and AST, as well as reduce hepatic lipid accumulation and ameliorate insulin sensitivity. Abbreviations: VEGF: vascular endothelial growth factor; TGF-*β*: transforming growth factor-*β*; IL-1Ra: interleukin-1 receptor agonist; ER: endoplasmic reticulum; ROS: reactive oxygen species; AKT: protein kinase B; AMPK: AMP-activated protein kinase; HFD: high-fat diet; GCK: glucokinase; L-PK: liver pyruvate kinase; PFK: 6-phosphofructo-1-kinase; PGC-1*α*: peroxisome proliferator *γ*-activated receptor coactivator 1-*α*; PEPCK: phosphoenolpyruvate carboxykinase; G6Pase: glucose-6-phosphatase; AKP: alkaline phosphatase; LDH: lactate dehydrogenase; ALT: alanine aminotransferase; AST: aspartate aminotransferase.

Moreover, MSCs demonstrate their repairing potential through the secretion of versatile cytokines and growth factors, including transforming growth factor- (TGF-) *β*, interleukin- (IL-) 6, and VEGF, which participate through both the paracrine and autocrine actions to enhance the islet function [52] and facilitate the vascularization process (Figure 1) [53]. In addition, some researchers correlated the islet repairing potential of MSCs to their antiapoptotic effects. Briefly, Borg et al. proved that BM-MSCs could reduce islet cell apoptosis as decreased cleavage of caspase 3 *in vivo* was observed after MSC treatment [54]. Chandravanshi and Bhonde further proved the antiapoptotic effect of MSCs by downregulating reactive oxygen species (ROS), nitric oxide, superoxide ions, caspase 3, caspase 8, and p53 and upregulating Bcl2 under hypoxia circumstances [55]. Besides, BM-MSCs are able to alleviate endoplasmic reticulum stress- (ERS-) induced apoptosis by overexpressing Myc through stromal cell-derived factor- (SDF-) 1 signaling or cell-cell interaction (Figure 1) [56].

Besides, MSCs are capable of enhancing the formation of autophagosomes by clearing impaired mitochondria and increasing the number of insulin granules (Figure 1) [22]. Mitochondria are key players in energy production, signaling, and apoptosis in cells, and their dysfunction has become the hallmark of various diseases, including diabetes, ischemia, inflammation, and aging. An increasing number of studies have revealed that MSC-mediated mitochondrial transfer is a mainstay to rescue injured cells and restore mitochondrial functions [57, 58]. Rackham et al. demonstrated that mitochondria of MSCs could be transferred to *β*-cells under hypoxia conditions for replenishment. Consequently, the oxygen consumption rate and insulin secretion rate of islet cells were enhanced after being cultured with MSCs, indicating that mitochondrial transfer could respond to and alleviate hypoxic and oxidative stress caused by excessive ROS production from damaged mitochondria [59]. Considering that mitochondria play a central role in energy metabolism, their intercellular transfer may partially explain the therapeutic mechanism of MSCs in improving *β*-cell regeneration. Besides, an increasing number of studies have postulated that mitochondrial donation by MSCs can also ameliorate other diabetic complications, including diabetic nephropathy and inflammation [58, 60, 61].

### 4.2. Hepatic Metabolic Homeostasis

T2DM is strongly associated with hepatic dysfunction, provided that around 57% to 80% of T2DM patients are suffering from MAFLD. In short, the relationship between MAFLD and T2DM is intricate and bidirectional, as they share similar features and metabolic syndromes, such as the accumulation of hepatic lipids, oxidative stress, and glucose tolerance [62]. In 2012, Ezquer et al. found that intravenously transplanted MSCs could significantly lower a panel of disordered biochemical markers of liver function caused by HFD, for instance, alkaline phosphatase (AKP), lactate dehydrogenase (LDH), alanine aminotransferase (ALT), and aspartate aminotransferase (AST), implying that MSCs could improve liver function of T2DM patients (Figure 1) [18].

PPARs are the major regulators of lipid metabolism, which help to control the balance of fatty acid uptake, adipogenesis, and *β*-oxidation. After MSC administration, PPAR-*α* was upregulated while PPAR-*γ* was downregulated in the liver of HFD mice, denoting that PPAR signaling pathways modulated by MSCs implicitly influence hepatic metabolism [17]. Besides, the expression of enzymes associated with hepatic glycolysis, including glucokinase (GCK), liver pyruvate kinase (L-PK), and 6-phosphofructo-1-kinase (PFK), was greatly elevated. Meanwhile, enzymes involved in gluconeogenesis, such as peroxisome proliferator *γ*-activated receptor coactivator 1-*α* (PGC-1*α*), phosphoenolpyruvate carboxykinase (PEPCK), and glucose-6-phosphatase (G6Pase), were reduced [24]. Evidence shows that the infusion of MSCs will activate protein kinase B (AKT) and AMP-activated protein kinase (AMPK) signaling pathways, which play indispensable roles in cell metabolism (Figure 1) [24, 25].

Furthermore, oxidative stress caused by mitochondrial dysfunction will also lead to liver metabolic imbalance [63]. The glutathione (GSH)/oxidized glutathione (GSSG) ratio was reduced, and the amount of superoxide dismutase, which is inversely proportional to systemic ROS levels, was increased after MSC treatment (Figure 1) [64, 65], postulating that the therapeutic effect of MSCs is highly associated with metabolic homeostasis. Meanwhile, treatment using an MSC-conditioned medium exhibited similar effects, suggesting that paracrine effects significantly contribute to the reparation process in T2DM [25]. In our recent work, we demonstrated that the intravenously injected MSCs resided in the liver on day 5 postadministration and persisted for 15 days. Besides, bioactive factors (e.g., IGFBP2 and APOM) secreted by MSCs paracrinely augmented insulin sensitivity and decreased lipid accumulation in hepatocytes through PI3K-AKT activation [30].

### 4.3. Alleviation of Insulin Resistance

**Figure 2:**
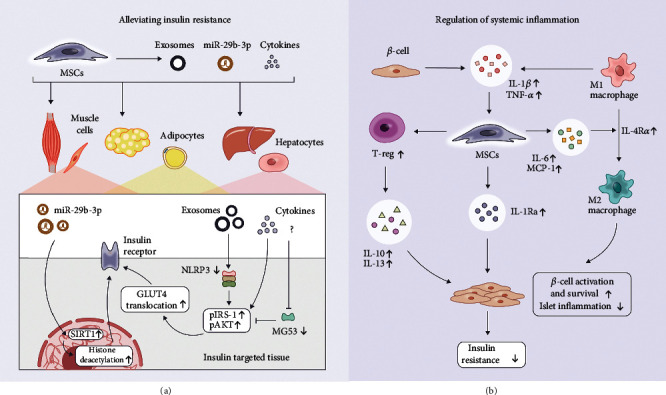
(a) Possible mechanisms of action for MSCs on insulin target organs to alleviate insulin resistance. Exosomal miR-29b-3p can regulate cellular insulin sensitivity via SIRT-1. Furthermore, NLRP3 formation can be inhibited through immune response regulation mediated by MSCs, thus enhancing the function of IRS-1 and GLUT4 in hepatic cells. MSCs also facilitate the inhibition of MG53, which is an E3 ligase that promotes the ubiquitinoylation of IRS-1 in skeletal muscles. (b) Possible mechanisms of action for MSCs to regulate systemic inflammation. IL-1*β* and TNF-*α* secreted by the T2DM islet will stimulate MSCs to secrete IL-1Ra, which in turn ameliorates islet inflammation. MSCs can also promote the proliferation of Treg cells, and IL-10 and IL-13 secreted by Treg seem to play a key role in islet regeneration by reducing systemic inflammation. Besides, classically activated macrophages (M1) could stimulate MSCs to overexpress IL-6 and MCP-1, thus converting M1 into an alternatively activated phenotype (M2) to reduce systemic inflammation. Abbreviations: SIRT-1: sirtuin-1; NLRP3: NOD-like receptor protein 3; IRS-1: insulin receptor substrate-1; GLUT4: glucose transporter 4; MG53: Mitsugumin 53; Treg: regulatory T; TGF-*β*: transforming growth factor-*β*; MCP-1: monocyte chemoattractant protein-1; IL: interleukin; TNF-*α*: tumor necrosis factor-*α*.

Insulin resistance, which is a distinctive hallmark of T2DM, describes the failure of cells to respond to insulin during disease progression. Lately, Si et al. revealed that intravenously injected BM-MSCs could increase GLUT expression and elevate phosphorylation of insulin receptor substrate-1 (IRS-1) and AKT in the target tissues of insulin [66], delineating that MSCs are capable of alleviating insulin resistance of diabetic patients. Furthermore, Deng et al. also showed that Mitsugumin 53 (MG53), an E3 ligase that promotes the ubiquitinoylation of IRS-1 in skeletal muscles, was inhibited by MSCs (Figure 2) [67]. Akin to the skeletal muscle that accounts for 70%-80% of insulin-stimulated glucose disposal, inhibition of the IRS-1 ubiquitin pathway may also engage in alleviating insulin resistance [68]. Moreover, insulin resistance in MAFLD and subsequent hepatic diseases is associated with the overproduction of inflammatory mediators and their downstream signaling molecules, with evidence suggesting that NOD-like receptor protein 3 (NLRP3) inflammasomes play an important role in obesity-induced insulin resistance [21]. The application of MSCs in T2DM treatment exemplifies that NLRP3 formation was inhibited through immune response regulation of MSCs, thus enhancing the function of IRS-1 and GLUT4 in hepatic cells (Figure 2) [35].

Besides, exosomes, which are nanoscale extracellular vesicles, also show broad prospects in tissue regeneration and damage reparation. *In vivo* experiments have further demonstrated the therapeutic effects of intravenously injected MSC exosomes in reducing the blood glucose level, as well as restoring the phosphorylation of IRS-1 and AKT signaling pathways in insulin target tissues [20]. The latest study confirmed that exosomal miR-29b-3p can regulate cellular insulin sensitivity via sirtuin- (SIRT-) 1 (Figure 2) [14], which is a class III histone deacetylase deeply involved in apoptosis, genomic stability, and gene expression regulation, indicating that histone modification related to insulin resistance is one of the treatment approaches of MSCs. Moreover, the clearance of dysfunctional mitochondria, alleviation of ERS, and diminishment of ROS may ameliorate insulin resistance [69].

### 4.4. Regulation of Systemic Inflammation

It is notorious that the pathogenesis of obesity-related insulin resistance includes chronic low-grade inflammation and activation of the immune system [21, 70]. Therefore, overexpression of systemic inflammatory cytokines, such as tumor necrosis factor- (TNF-) *α*, interleukin- (IL-) 1*β*, and IL-6, is accompanied by the pathogenesis of metabolic syndromes, including insulin resistance, atherosclerosis, and MAFLD (Figure 2). Likewise, the abnormal changes in peripheral or tissue-resident immune cells and their regulatory function always accompany the development of diabetes, indicating that immune cells such as T lymphocytes (T cells), macrophages, and natural killer cells (NK cells) are considered to participate in the progression of T2DM concomitantly [21].

It has been a prevailing dogma that MSCs have immune privilege properties. This is exemplified by the immunomodulatory effects of MSCs on T cells, B lymphocytes (B cells), dendritic cells (DCs), and NK cells, mainly via paracrine effects that involve the secretion of enzymes, chemokines, cytokines, anti-inflammatory mediators, growth factors, and extracellular vesicles [71, 72]. Briefly, MSC activation is subjected to the stimulation of a multitude of inflammatory cytokines, including TNF-*α* and interferon- (IFN-) *γ*, which in turn shift to an immunosuppressive phenotype by inducing the secretion of soluble factors that mediated immunomodulatory activities, such as prostaglandin E2 (PGE2), hepatocyte growth factor (HGF), indoleamine-pyrrole 2,3-dioxygenase (IDO), and IL-10 [73, 74]. Additionally, the paracrine immunomodulatory properties of MSCs are highly mediated by versatile signaling pathways like the telomerase-associated protein Rap1/NF-*κ*B pathway [75]. Although we do not have a comprehensive understanding of the precise mechanism of MSC-based immunomodulation, MSCs have been harnessed for the treatment of immune-mediated disorders [76, 77], including graft-versus-host disease (GvHD) and diabetes.

To date, experimental results showed that the inflammatory status of STZ-diabetic animal models contributes to the modification of the pancreatic microenvironment, while the administration of MSCs promoted the proliferation of regulatory T cells (Tregs) to provide long-term immunoregulatory effects [78]. Consequently, Th2 cytokines (IL-10 and IL-13) secreted by Tregs seem to play a key role in *β*-cell activation and survival through their anti-inflammatory effects, where the definitive mechanism of action remains to be an enigma (Figure 2) [19]. Besides, the mobilization of MSCs by inflammatory factors under specific microenvironments has been demonstrated, illustrating that MSCs can elicit the transition of macrophages into an anti-inflammatory phenotype to alleviate insulin resistance in T2DM rats [34, 36]. In brief, classically activated macrophages (M1) could stimulate MSCs to overexpress IL-6 and MCP-1, thus converting M1 into an alternatively activated phenotype (M2) (Figure 2). Meanwhile, IL-4R expression was upregulated in macrophages, which sensitizes them to the IL-4 stimulus. Moreover, MSCs can downregulate the systemic inflammatory cytokines to impair insulin receptor action and respective downstream signaling pathways by preventing the formation of NLRP3 in the adipose tissue and liver [35]. Wang et al. demonstrated that IL-1*β* and TNF-*α* secreted by the T2DM islet could stimulate MSCs to secrete IL-1Ra, which could ameliorate islet inflammation (Figure 2) [37]. In conclusion, the above mechanistic investigation provides a theoretical basis for the clinical application of MSCs in the treatment of T2DM along with its associated complications.

## 5. Pharmacokinetics of MSCs in T2DM

Although MSCs have shown their potential in treating T2DM both *in vitro* and *in vivo*, we have not thoroughly understood their *in vivo* behavior, which hampers further progress for clinical investigation in the field of MSC-based T2DM therapy [79]. It is generally known that there is often a discrepancy in the kinetics of MSCs among different cell sources, T2DM models, and routes of administration [80]. Therefore, the ability to determine the dose, *in vivo* distribution, and extended viability of MSCs in patients is crucial in developing MSC-based therapies and elucidating the *in vivo* therapeutic mechanism of administered MSCs for T2DM treatment [81]. Furthermore, increased knowledge of MSC distribution after delivery could allow researchers to estimate cellular pharmacokinetics, thus identifying the dosing scheme required to achieve optimal therapeutic effects [82]. Akin to the use of a PK model for drug development, which delineated the time course of drug absorption, distribution, metabolism, and excretion (ADME), an effective *in vivo* kinetic model of administered MSCs and their released factors should be adapted and applied to allow clinical translation of therapeutic MSCs in treating T2DM. If robust pharmacokinetic models of MSCs can be developed, the therapeutic efficacy of MSCs in various treatment conditions can be predicted, thus informing the optimal administration regimes of the cells and hastening the progression of clinical research [80].

### 5.1. *In Vivo* Kinetics of Systematically Applied MSCs

Despite the rapid progress in using MSCs as a safe and effective treatment of T2DM, the *in vivo* PK of administered MSCs is rarely reported. Sood et al. labeled BM-MNCs with a positron emission tomography (PET) tracer, namely, fluorine 18-fluorodeoxyglucose (^18^F-FDG), to track the biodistribution of cells *in vivo*. BM-MNCs were administered to diabetic patients through three different routes—peripheral intravenous, superior pancreaticoduodenal artery, and splenic artery injection—with the *in vivo* biodistribution of cells tracked and quantified at 30 and 90 minutes after administration. More BM-MNCs were retained in the pancreas after being administered through the superior pancreaticoduodenal artery, while no discernible cell was observed after splenic artery and intravenous injection. Besides the pancreas, the spleen also showed an intense FDG signal after splenic artery injection. On the contrary, the lung showed retention of cells within 30 minutes, with a significant clearance in 90 minutes after intravenous injection [83]. The study by Sood et al. did not track the BM-MNCs for a longer time. Furthermore, Yaochite et al. generated adipose-derived- (AD-) MSCs^Luc+^ that expressed luciferase and administered the cells to STZ-induced diabetic mice through intrasplenic or intrapancreatic injection. Following intrasplenic transplantation, AD-MSCs^Luc+^ were mainly observed in the liver and pancreas until day 8 after intrasplenic and intrapancreatic injection, respectively. However, these injection routes are rarely used in clinical trials, denoting that the long-term distribution of MSCs after intravenous injection should be further compared with the above administration routes in diabetic mice and patients [84].

### 5.2. Modeling the *In Vivo* Kinetics of MSCs

**Figure 3:**
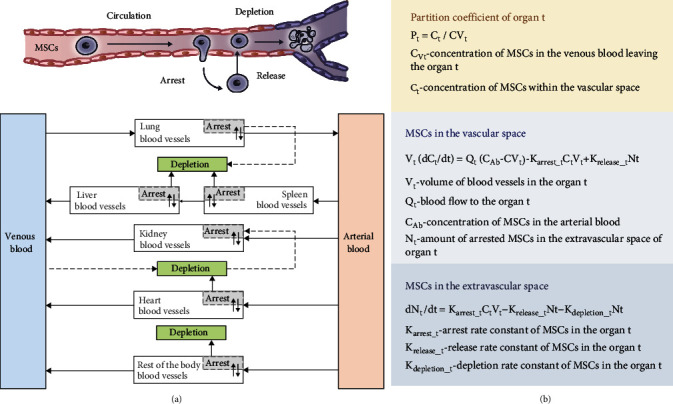
Structures and the mathematical equations of the available PK model of MSCs. (a) Schematic diagram of the PK model for the administered MSCs *in vivo*. Solid arrows indicate the blood flow, dashed grey arrows indicate the depletion of MSCs, and grey boxes indicate the arrested MSCs isolated from blood circulation as in the extravascular space of the organ. (b) The equations to calculate MSC concentration in the respective organs [79].

Although the biodistribution of cells can be quantified by various experiment techniques, the PK of administered cells has not been studied systematically through a PK model. During the past 30 years, many PK models have been developed to describe the ADME of conventional drugs, which were successfully applied to predict the safety and efficacy of therapeutic agents, including biologics and small-molecule drugs [80]. Studying the PK aspects of MSCs is difficult but critical in the development of MSC therapy, which could assist in the optimization of the cell dosage, mode of injection, course treatment, and targeting strategies to achieve maximum efficiency with the lowest risk [85]. To simplify the explanation of the *in vivo* kinetics of therapeutic cells, the dynamics of systematically administered cells have been considered similar to those of inert micrometer-scale particles injected into the bloodstream of animals [83, 86, 87]. To date, the only published PK model of MSCs was developed in 2016 [79]. Wang et al. established a physiological-based (PB) PK model based on the anatomical structure of the body, which separates every important organ in the body as an individual compartment, and each of them is interconnected by blood vessels. In this simplified model, the whole body was divided into eight interconnected compartments, which were the arterial blood, lungs, liver, spleen, kidneys, heart, venous blood, and the rest of the human body (Figure 3) [37]. Once administered intravenously, most of the MSCs were rapidly transferred to the blood vessels of each organ through systemic blood flow. MSCs that reached the organs were either passively entrapped in the microvessels or actively adhered to the endothelial cells. The entrapped MSCs were either released back into the blood circulation or eliminated after depletion (Figure 3). Therefore, *K*_arrest_, *K*_release_, and *K*_depletion_ were used to represent the rate constants of the arrest, release, and depletion processes, respectively, along with other key parameters, including species-specific physiological parameters (body weight, organ volume, and blood flow) and MSC-specific parameters (partition coefficient, arrest rate constant, release rate constant, and depletion rate constant). Through this PBPK model, the time course of MSC concentration in blood and individual organs can be predicted across species, such as mice, rats, and humans. However, the model only predicts a fast distribution process of MSCs in the body within 24 hours, implying that optimization of the current model is imperative, as the slow biological process such as proliferation, senescence, and differentiation of the arrested MSCs should be incorporated into the model [88].

Besides, it is well established that MSCs can play their therapeutic roles beyond what is conveyed by the transplanted cells alone, mainly through the secretion of bioactive products, namely, the secretome [89]. Therefore, the PK of these factors, which are constantly secreted by MSCs, should be considered in a similar way to common pharmaceutical drugs [80]. Salvadori et al. used the approach described by Parekkadan and Milwid [86] to establish a new pharmacokinetic-pharmacodynamic (PK-PD) model, namely, the “two-functional-compartments PK-PD model” [82]. In this model, the cell-related biomarkers released by MSCs, which are capable of influencing bystander cells (e.g., macrophages), can secrete specialized bioactive substances that play a main role in the PD of administered cells [90]. Accordingly, they described that MSCs can attenuate sepsis by releasing PGE2, which binds to PGE2 receptors of activated macrophages and provokes the release of IL-10 that in turn reduces inflammation by acting on immune cells [86]. Moreover, other supporting data on this concept have been reported [91]. Nevertheless, the present models are still unable to represent the long-term *in vivo* kinetics of MSCs and their secretomes adequately.

In short, understanding the *in vivo* kinetics of administered drugs can be challenging, especially for nontraditional drugs such as MSCs. A functional PK-PD model may begin to predict the pharmacokinetics of MSC therapies through a specific formulation and administration pathway by utilizing both *in silico* modeling and empirical analysis. Besides, studies of the pharmacokinetic model have the ability for interspecies scaling, allowing us to predict the *in vivo* kinetics of therapeutic MSCs in humans through animal data. However, it is indefinite how well the findings in animals can be quantitatively transferred to humans. Despite the use of MSCs in clinical trials, many details still need to be discussed as their biodistribution varies under different treatment conditions. Therefore, combined PK-PD modeling describing both the biodistribution and the functional secreted factors of MSCs should be unraveled to achieve more efficacious MSC therapeutics in the future.

## 6. Conclusions and Future Perspectives

According to the summarization of preclinical and clinical results in the aforementioned studies, MSC-based therapy has made tremendous progress in T2DM treatment in both animal studies and clinical trials. Aside from the necessity of developing a robust PK-PD model, there are still many encumbrances for MSCs to transit out of the laboratory stage and be launched as therapeutic products in the pharmaceutical industry. Some of these impediments have become a mutual subject in the field of cell therapy, but some necessitate particular considerations due to the special characteristics of T2DM that are distinct from other diseases. Therefore, various challenges in the clinical development of MSC therapy for T2DM are discussed here, which include but are not restricted to the limited therapeutic effects caused by the lung barrier, the capillary blockage caused by microthrombus, and the selection of diverse cell sources.

### 6.1. Potential Risks of Intravenously Administered MSCs

The delivery routes of MSCs have been shown to dramatically influence the therapeutic effects of MSCs. In small animal and clinical studies, intravenous injection is the most frequently used administration route and the bioluminescence imaging system is widely accepted to track the *in vivo* biodistribution of MSCs. Impoverished cell survival was discovered as most of the MSCs were trapped within the lung capillaries and eliminated within hours posttail vein injection [92]. Still, the fate of MSCs in the lung is controversial as the fluorescence signal gradually disappeared during long-term tracking [93]. Furthermore, microthrombus that contributes to the blockages in lung capillaries also arises as a potential safety issue in cell therapy. According to previous studies, intravenous infusion of MSCs will lead to a reduced blood flow velocity in the lung capillaries, which resulted in the formation of local thrombus in the blood vessels [94]. In order to resolve this drawback, heparin was mixed with cell suspension by Liao et al. during systemic injection [95], while MSCs were pretreated with hypertonic solution by Leibacher et al. to reduce the cell size [96]. Besides, Leibacher et al. also suggested that the size of MSCs would gradually increase with prolonged culture passage [96], indicating that the infusion of MSCs with lower passage will reduce the formation of microthrombus.

### 6.2. MSC Sources in Clinical Applications

Although MSCs derived from various sources such as the umbilical cord, adipose tissue, and bone marrow have shown efficacy in relieving T2DM in preclinical and clinical studies, the clinical success of MSC therapy is still facing great challenges due to their compromised expansion potential and age-associated functional decline, as well as the setbacks in the standardized and large-scale manufacture of therapeutic MSCs [97, 98]. Primary MSCs isolated from different donors, tissue sources, cell separation methods, or culture conditions show natural heterogenicity, which causes batch-to-batch variation and diverse differential and therapeutic efficacy [97, 98]. Therefore, the production of MSCs complying with the current good manufacturing practice (cGMP) standards becomes a prerequisite to ensure the standardization and reproducibility, as well as the quality and safety of MSCs for clinical use [99]. Besides, although MSCs have been considered safe with minimal tumorigenicity after transplantation, genetically modified MSCs are facing safety concerns, including the immunogenic toxicity of viral vectors, insertional oncogenesis, and mutational integration [97].

To date, there are insufficient studies that have compared the therapeutic effects of MSCs from different sources and engineering methods systematically. Therefore, the selection of MSCs that can achieve the best prognosis effect under diverse clinical circumstances remains an arduous task to be investigated concertedly. Particularly, the ability of AD-MSCs to improve glucose tolerance effectively is evidenced by both animal studies and preclinical studies. However, despite the lower production cost, higher feasibility, and superior *in vitro* expansion ability of AD-MSCs, research shows that adipose tissue in T2DM patients is in an inflammatory state and is accompanied by a certain degree of cell aging [100]. Aging cells will disrupt tissue function through their senescence-associated secretory phenotype (SASP), which contains a large number of inflammatory factors, thus contributing significantly to systemic inflammation in T2DM patients [101]. Furthermore, SASP can cause insulin resistance in liver cells and apoptosis of islet cells, given that transplanting senescent adipose tissue will affect the animal's behavioral ability and accelerate the aging of mice [102]. Therefore, it is still questionable whether autologous AD-MSCs are suitable for the treatment of T2DM, concerning that the adipocytes in diabetic patients are in an inflammatory state. Besides, autologous cells are hard to be developed as off-the-shelf products due to their longer processing duration after being extracted from patients. On the other hand, although studies have shown that MSCs exhibit immunoregulatory effects, it remains elusive as to what degree the allogeneic cells trigger immune responses *in vivo* after the administration [103].

Aside from primary tissue-derived MSCs, the employment of MSCs differentiated from human pluripotent stem cells (PSCs), including embryonic stem cells (ESCs) and induced pluripotent stem cells (iPSCs), may potentially be more desirable choices as safer and more effective MSC medications against T2DM [104]. In particular, iPSCs based on cell reprogramming technology have provided unprecedented opportunities to expedite the development of human cell therapies, without involving the ethical issues of ESCs. Briefly, the advantages of iPSC-derived MSCs (iMSCs) include their potential to produce infinite donor-related sources of specific stem cells with improved homogeneity, stability, controllability, and scalability, thus becoming a preferential commercial candidate for clinical applications [97, 98]. Besides, it is increasingly appreciated that human iMSCs exhibit higher proliferative potential and display potent immunomodulatory properties [105, 106]. To date, Cymerus™ MSCs (CYP-001), which are derived from adult iPSCs produced by an optimized GMP-compliant manufacturing process, have been characterized by Cynata Therapeutics and received approval to launch the world's first formal trial for the treatment of acute steroid-resistant GvHD [107, 108]. However, it is worth noting that there are still a few hurdles, such as potential tumorigenicity, immunogenicity, and heterogeneity, which remain to be overcome when using iMSCs for downstream applications, including T2DM therapy in the future [104].

### 6.3. Unidentified Therapeutic Mechanisms of MSCs *In Vivo*

Although the therapeutic success is substantiated by MSCs in preclinical and clinical studies, their mechanisms of action in the progression of T2DM become the foremost issue to be thoroughly elucidated. Meanwhile, although researchers reach a consensus on the immunosuppressive effect of MSCs [32], the mechanism that describes how MSCs can affect systemic inflammation has not been thoroughly clarified. Galleu et al. investigated the therapeutic effects of MSCs on GvHD and suggested that those MSCs that resided in the lung were attacked by cytotoxic T cells and NK cells, thus leading to cell apoptosis [109]. Consequently, fragments produced by apoptotic MSCs are phagocytosed by macrophages to produce indoleamine 2,3-dioxygenase, which helps to mediate systemic inflammation inhibition. However, it remains unclear whether apoptotic MSCs contribute to the same therapeutic mechanism in treating T2DM. In addition, Akiyama et al. believe that MSCs can induce T cell apoptosis through their production of the Fas ligand, and the apoptotic fragments are swallowed by macrophages to trigger systemic immune regulation [110], suggesting that the diverse roles of MSCs might lead to potential safety risks in clinical use. Therefore, uncovering the fate of MSCs *in vivo* will pave the way for the understanding of therapeutic mechanisms to accelerate the progress of their clinical translation.

Despite the complexity involved in the pathological process of diabetes, various conventional drugs are capable of lowering blood sugar levels through different mechanisms. However, it is uncertain whether antidiabetic drugs can reverse the pathological progression of T2DM. Thiazolidinedione, which possesses the potential to increase liver insulin sensitivity, has a risk of inducing heart failure or hepatic dysfunction [111]. GLP-1RAs have shown a broad range of therapeutic effects in various diseases aside from their antidiabetic effects. Recent studies have proved that liraglutide, exenatide, and semaglutide show promises in treating cardiovascular diseases [112, 113], MAFLD [114], obesity [115], ischemic stroke [112, 116, 117], Parkinson's disease [118, 119], and Alzheimer's disease [120]. Therefore, combinatory administration of GLP-1RAs and MSCs is expected to augment the therapeutic efficacy of both the antidiabetic drugs and the MSCs.

In conclusion, the therapeutic effects of MSCs on T2DM are multifaceted [121] and the possible therapeutic mechanisms have been summarized here. MSCs can improve the systemic inflammatory state through their immunosuppressive functions, reduce the apoptosis of islet *β*-cells to augment insulin secretion, and improve the metabolic state of the liver. However, further in-depth clarifications regarding the mechanisms of action of MSCs in treating T2DM are still a requisite. Therefore, whether it is possible for MSCs or novel MSC-assisted therapeutics to surpass traditional medicines in reversing the progression of T2DM remains an engrossing question to be explored in the future.

## Figures and Tables

**Table 1:** Summary of preclinical studies using MSCs to treat animals with diabetes mellitus.

Model/year	MSC sources	MSC dosage (cells)	Interventions	Effectiveness	References
HFD mice 2011	mBM-MSCs	5 × 10^5^	Intravenous, single injection	Prevented the onset of nonalcoholic steatohepatitis in obese mice with metabolic syndromes	Ezquer et al. [18]
HFD mice 2014	rBM-MSCs	1 × 10^4^/g of body weight	Intravenous, single injection	Ameliorated diabetic hepatocyte damage by inhibiting infiltration of bone marrow-derived cells	Nagaishi et al. [25]
Fat-fed/STZ rat 2015	rBM-MSCs	2 × 10^6^	Intravenous, single injection	Ameliorated chronic high glucose-induced *β*-cell injury through modulation of autophagy	Zhao et al. [22]
Fat-fed/STZ rat 2015	hUC-MSCs	3 × 10^6^	Intravenous, single injection	Elicited macrophages into an anti-inflammatory phenotype to alleviate insulin resistance	Xie et al. [34]
HFD mice 2016	hAD-MSCs	1 × 10^6^	Intraperitoneal, once every 2 weeks (up to 10 weeks)	Reduced obesity and metabolic syndromes	Lee et al. [17]
Fat-fed/STZ rat 2016	mAD-MSCs	3 × 10^6^	Intravenous, single injection	Ameliorated hyperglycemia through regulating hepatic glucose metabolism	Xie et al. [24]
Fat-fed/STZ rat 2017	hUC-MSCs	3 × 10^6^	Intravenous, single injection	Ameliorated insulin resistance by suppressing NLRP3 inflammasome-mediated inflammation	Sun et al. [35]
db/db mice Fat-fed/STZ 2018	mAD-MSCs	5 × 10^5^	Intravenous, single injection	Improved insulin sensitivity and less *β*-cell death seen in T2DM mice	Wang et al. [23]
Fat-fed/STZ rat 2018	hUC-MSCs	hUC-MSCs: 3 × 10^6^ Exosomes: 10 mg/kg	(1) Cell injection: intravenous, once every 2 weeks (up to 10 weeks) (2) Exosome injection: intravenous, once every 3 days for 5 cycles	Exosomes from hUC-MSCs could alleviate T2DM by reversing peripheral insulin resistance and relieving *β*-cell destruction	Sun et al. [20]
Fat-fed/STZ rat 2018	hUC-MSCs	1 × 10^6^	Intravenous, single injection	Directed macrophage polarization to alleviate pancreatic islet dysfunction	Yin et al. [36]
db/db mice 2019	hUC-MSCs	1 × 10^6^	Intravenous, once at 7 and 9 weeks of age	Reversed *β*-cell dedifferentiation in an IL-1Ra-mediated manner	Wang et al. [37]
HFD mice 2021	hAD-MSCs	1 × 10^6^	Intravenous, once every 2 weeks (up to 1 month)	Achieved MSC self-augmentation, suppressed senescence and apoptosis of pancreatic *β*-cells, and decreased lipid accumulation in hepatocytes	Zhang et al. [30]

Abbreviations: BM-MSCs: bone marrow mesenchymal stem cells; UC-MSCs: umbilical cord mesenchymal stem cells; AD-MSCs: adipose-derived mesenchymal stem cells; STZ: streptozotocin; NLRP3: NOD-like receptor protein 3; IL-Ra: interleukin receptor agonist.

**Table 2:** Summary of clinical trials using MSCs to treat patients with T2DM.

Year/sample size	MSC sources	MSC dose (cells)	Interventions	Outcome measurements	References/NCT number
2008 *N* = 25	BM-SCs	Unknown	Intrapancreatic injection	Fasting glucose, HbA1c, and insulin requirements decreased; fasting C-peptide and C-peptide/glucose ratio increased	Estrada et al. [38]
2014 *N* = 21	Autologous BM-SCs	3.1 × 10^6^ cells/kg	Intrapancreatic injection	The insulin requirement decreased; HbA1c increased modestly and nonsignificantly; glucagon-stimulated C-peptide increased significantly	Bhansali et al. [41]
2016 *N* = 31	WJ-MSCs	1 × 10^6^ cells/kg	Two intravenous infusions, with a one-month interval	No serious adverse reactions; improvements in C-peptide and insulin dosage in the MSC group	Hu et al. [42]
2011 *N* = 10	Allogeneic HP-MSCs	1.35 × 10^6^ cells/kg	Three intravenous infusions of PDSCs, with a one-month interval	No acute adverse events, average insulin dosage, C-peptide, and HbA1c improved after treatment	Jiang et al. [16]
2010 *N* = 22	Allogeneic WJ-MSCs	1 × 10^6^ cells/kg	Infused into the peripheral vein on day 5; delivered directly to the pancreas via he splenic artery using endovascular catheters on day 10	Fever, subcutaneous hematoma, and headache were observed; mild improvement in HbA1c, insulin dosage, and fasting C-peptide; markers of systemic inflammation were decreased	Liu et al. [40]
2014 *N* = 30	UC-MSCs	1 × 10^6^ cells/kg	UC-MSCs were intravenously transfused three times. All patients were followed up in the first, third, and sixth months	FBG and PBG were significantly reduced; plasma C-peptide levels and regulatory T cell numbers were numerically higher	Kong et al. [43]
2014 *N* = 80	BM-MNCs	3.8 × 10^9^ cells/kg	Pancreatic artery infusion in 10 min	No acute adverse events; the area under the curve of C-peptide was significantly improved	Wu et al. [44]
2015 *N* = 61	Allogeneic BM-MSCs	0.3-2 × 10^6^ cells/kg	Subjects were randomized to receive one of the following three rexlemestrocel-L doses or placebo in a 3 : 1 ratio using a sequential, escalating dose cohort paradigm	No acute adverse events; HbA1c was reduced at all time points after week 1	Skyler et al. [45]
2016 *N* = 30	Allogeneic BM-MSCs	150-300 × 10^6^ cells/kg	Treatment was administered by intravenous infusion on day 0 following baseline assessments; two sequential dose cohorts, to receive rexlemestrocel-L or placebo; study duration was 60 weeks	No acute adverse events; improved eGFR and mGFR at week 12	Packham et al. [46]
2016 *N* = 100	UC-MSCs	1 × 10^6^ cells/kg	The hUC-MSCs were transplanted by infusing 1 × 10^6^ cells/kg via the pancreatic artery directed on day 1, followed by infusing 1 × 10^6^ cells/kg through the peripheral vein on days 8, 15, and 22	*Δ*CP30/*Δ*G30 and AUCCP180 increased; FPG, 2hPG, HbA1c, and HOMA-IR reduced	Li et al. [39]
2017 *N* = 30	Autologous BM-MSCs and autologous BM-MNCs	MSCs: 1 × 10^6^ cells/kg; MNCs: 10^9^ per patient	Intrapancreatic infusion	Significant reduction in insulin requirement; significant increase in the second-phase C-peptide response and insulin sensitivity index	Bhansali et al. [47]
August 2010 *N* = 24	BM-MSCs	Unknown	BM-MSCs are transplanted through the pancreatic artery percutaneously on day 0; BM-MSCs are transplanted intravenously on days 7 and 14	Unknown	NCT01142050
January 2011 *N* = 22	Autologous BM-MNCs Autologous BM-MSCs	Unknown	Three groups treated with BM-MSCs, BM-MNCs, and insulin	Unknown	NCT01719640
January 2013 *N* = 200	UC-MSCs	Intravenous/infusion treatment	Unknown	Unknown	NCT02302599
September 2015 *N* = 30 Recruiting	Injectable collagen scaffold with hUC-MSCs	1.5 × 10^7^ intracavernous injection	Intracavernous injection of an injectable collagen scaffold combined with 15 million hUC-MSCs; intracavernous injection of 15 million hUC-MSCs	Unknown	NCT02745808
November 2017 *N* = 30	Autologous BM-MSCs	Unknown	Pancreatic artery infusion	Unknown	NCT03343782
April 2019 *N* = 60 Recruiting	Autologous BM-MNCs and allogeneic UC-MSCs	UC-MSC: 1-2 × 10^6^ cells/kg intravenous infusion	Unknown	Unknown	NCT03943940
November 2019 *N* = 54 Recruiting	hUC-MSCs	1.5 × 10^6^ cells/kg peripheral intravenous infusion	Unknown	Unknown	NCT04216849

Abbreviations: BM-SCs: bone marrow stem cells; WJ-MSCs: Wharton's jelly-derived mesenchymal stem cells; UC-MSCs: umbilical cord mesenchymal stem cells; BM-MNCs: bone marrow mononuclear cells; BM-MSCs: bone marrow mesenchymal stem cells; HP-MSCs: hypoxia preconditioned mesenchymal stem cells; PDSCs: placenta-derived stem cells; FBG: fasting blood glucose; PBG: postprandial blood glucose; eGFR: estimated glomerular filtration rate; mGFR: measured glomerular filtration rate; 2hPG: postload glucose; FPG: fasting plasma glucose; AUCCP180: amount of C-peptide secretion function; HbA1c: hemoglobin A1c; HOMA: homeostatic model assessment; IR: insulin resistance.

## Data Availability

The references used to support the findings of this study are included within the article.
